# Ablation of the androgen receptor from vascular smooth muscle cells demonstrates a role for testosterone in vascular calcification

**DOI:** 10.1038/srep24807

**Published:** 2016-04-20

**Authors:** Dongxing Zhu, Patrick W. F. Hadoke, Junxi Wu, Alex T. Vesey, Daniel. A. Lerman, Marc R. Dweck, David E. Newby, Lee B. Smith, Vicky E. MacRae

**Affiliations:** 1 The Roslin Institute and Royal (Dick) School of Veterinary Studies, University of Edinburgh, Midlothian, EH25 9RG, UK; 2 BHF Centre for Cardiovascular Science, University of Edinburgh, The Queen’s Medical Research Institute, 47 Little France Crescent, Edinburgh, EH16 4TJ, UK; 3 MRC Centre for Reproductive Health, University of Edinburgh, The Queen’s Medical Research Institute, 47 Little France Crescent, Edinburgh, EH16 4TJ, UK; 4 MRC Centre for Regenerative Medicine, University of Edinburgh, 5 Little France Drive, Edinburgh, EH16 4UU, UK; 5 The Royal Infirmary of Edinburgh Hospital: Royal Infirmary of Edinburgh, 51 Little France Crescent, Old Dalkeith Road, Edinburgh, EH16 4SA, UK

## Abstract

Vascular calcification powerfully predicts mortality and morbidity from cardiovascular disease. Men have a greater risk of cardiovascular disease, compared to women of a similar age. These gender disparities suggest an influence of sex hormones. Testosterone is the primary and most well-recognised androgen in men. Therefore, we addressed the hypothesis that exogenous androgen treatment induces vascular calcification. Immunohistochemical analysis revealed expression of androgen receptor (AR) in the calcified media of human femoral artery tissue and calcified human valves. Furthermore, *in vitro* studies revealed increased phosphate (Pi)-induced mouse vascular smooth muscle cell (VSMC) calcification following either testosterone or dihydrotestosterone (DHT) treatment for 9 days. Testosterone and DHT treatment increased tissue non-specific alkaline phosphatase (*Alpl*) mRNA expression. Testosterone-induced calcification was blunted in VSMC-specific AR-ablated (SM-ARKO) VSMCs compared to WT. Consistent with these data, SM-ARKO VSMCs showed a reduction in *Osterix* mRNA expression. However, intriguingly, a counter-intuitive increase in *Alpl* was observed. These novel data demonstrate that androgens play a role in inducing vascular calcification through the AR. Androgen signalling may represent a novel potential therapeutic target for clinical intervention.

Being male is associated with an increased risk of cardiovascular disease^1^, suggesting that androgens may negatively impact the cardiovascular system. However, recent studies have suggested that testosterone may in fact exert protective effects on the cardiovascular system, as low plasma testosterone levels are associated with advanced atherosclerosis and independently related to cardiovascular disease and mortality^2^,^3^,^4^,^5^,^6^. Understanding this apparent discrepancy is of paramount importance, given the dramatic increase in the use of Androgen Replacement Therapy (ART) in men over the last decade^7^,^8^. Furthermore, the continued abuse of anabolic androgens in sport^9^ induces long term adverse medical effects including cardiovascular toxicity along with manic behavioural symptoms^10^.

Testosterone is the primary androgen in men. The biological actions of testosterone, and particularly of nonaromatizable dihydrotestosterone (DHT), are predominantly mediated by the androgen receptor (AR)^11^. The AR modulates transcription by binding to androgen-response elements (AREs) in the promoter and enhancer regions of target genes in the nucleus^12^. The AR is expressed throughout the arterial wall^13^,^14^ and has been reported to play a notable role in vascular disease^15^,^16^. Additionally, testosterone can be converted to estradiol by aromatase expressed in the vessel wall^17^. The induction of rapid, non-genomic actions through interactions with additional signalling pathways may further refine the cellular effects of testosterone^12^,^18^.

Vascular calcification is a life-threatening complication of cardiovascular disease, affecting tissues including arteries, heart valves and cardiac muscle^19^,^20^,^21^,^22^. Whilst the mechanisms underpinning the pathogenesis of calcified aortic valve disease (CAVD) are still unclear, it has been shown to share many features with the predominantly studied arterial calcification^22^. Both CAVD and arterial calcification are recognised as active, tightly regulated processes, sharing many similarities with physiological bone formation^23^ and involves the deposition of hydroxyapatite crystals in arteries. Indeed VSMCs, the predominant cell type involved in vascular calcification, can undergo transdifferentiation to a chondrocytic, osteoblastic and osteocytic phenotype in a calcified environment^24^,^25^. Furthermore, it has been demonstrated that phosphate (P_i_) accelerates this phenotypic trans-differentiation, evident in the loss of characteristic smooth muscle markers and the development of osteoblastic features, such as the expression of tissue-nonspecific alkaline phosphatase (TNAP), sodium-dependent phosphate co-transporter (P_i_T-1), osteocalcin and osteopontin, and osteocyte markers including sclerostin and podoplanin (Pdpn/E11)^25^,^26^. Vascular calcification also involves the reciprocal loss of recognised calcification suppressors, such as inorganic pyrophosphate (PP_i_), matrix Gla protein (MGP) and fetuin A^27^,^28^.

The prevalence of vascular calcification is sexually dimorphic, showing a relative increase in men compared to women, which suggests possible hormonal influence^29^. Androgens have been reported to play a major regulatory role in bone formation, having significant effects on both osteoblast and osteoclast function^30^. The pro-calcificatory effects of androgens on established atherosclerotic lesions have recently been demonstrated in the Apolipoprotein E-null mouse model^31^. However, subsequent *in vitro* studies have suggested an inhibitory effect of testosterone on vascular calcification^32^. Given these inconsistent findings, there is a clear need for additional studies to firmly establish the role of androgens in vascular calcification. We have therefore undertaken analysis of clinical tissues in conjunction with *in vitro* calcification studies in C57BL/6 and SM-ARKO mice to address the hypothesis that testosterone induces vascular calcification through stimulation of AR.

## Results

### Expression of AR in human calcified cardiovascular tissue

**Figure 1:**
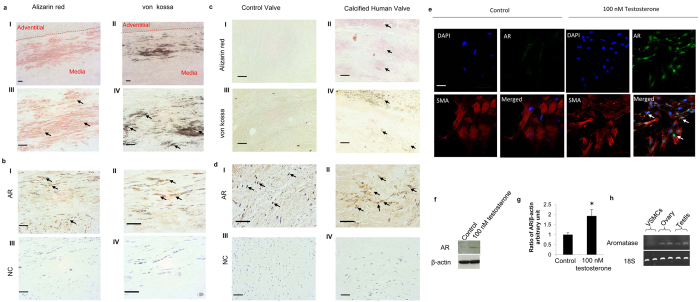
Androgen receptor (AR) expression in calcified human cardiovascular tissue and primary murine VSMCs. (**a**) Calcified human femoral artery was confirmed by alizarin red (I & III, arrows indicate positive alizarin red staining) and von kossa staining (II & IV, arrows indicate positive von kossa staining). (**b**) AR expression was observed in the calcified tunica media region of human femoral artery tissue (I & II, arrows indicate positive AR staining). (**c**) Calcified aortic valve was confirmed by alizarin red (I & II, arrows indicate positive alizarin red staining) and von kossa staining (III & IV, arrows indicate positive von kossa staining). (**d**) Higher AR expression was observed in calcified aortic valve compared to control (I & II, arrows indicate positive AR staining). (**e**) Immunofluorescence staining showed nucleus staining of AR in murine VSMCs (SMA-red, AR-green, DAPI-blue, arrows indicate colocalisation of AR and DAPI staining). (**f**) Representative image of western blotting for AR following 100 nM testosterone treatment in VSMCs (Images were cropped from original scans, and gels were performed under the same experimental conditions. Unprocessed original scans are shown in Suppl Fig. 1) and (**g**) densitometry quantification (n = 3) showed increased expression of AR in VSMCs following treatment with testosterone. (**h**) Aromatase expression was absent from murine VSMCs (Images were cropped from original scans, and gels were performed under the same experimental conditions. Unprocessed original scans are shown in Suppl Fig. 1). NC=Negative Control. Scale bar = 100 μm. *P < 0.05.

To investigate the role of the AR in vascular calcification, a critical and advanced phenotype of atherosclerosis, localisation studies were undertaken. Calcification of human femoral artery and aortic valve tissues was confirmed by alizarin red and von kossa staining (Fig. 1a I-IV,c I-IV). Immunohistochemistry established AR expression in calcified areas of human femoral artery and arterial valve tissues (Fig. 1b I-II,d I-II). Additionally, higher expression of AR was observed in calcified human aortic valve compared to control valve tissue (Fig. 1d I-II). These data are the first to show that AR is expressed alongside calcification in human calcified cardiovascular tissue.

Isolated VSMCs were positively stained with alpha smooth muscle actin (SMA) antibody (Fig. 1e, red). AR expression was confirmed in cultured murine VSMCs (the predominant cell type responsible for blood vessel calcification) by immunofluorescence staining (Fig. 1e, green), and was shown to increase following incubation with testosterone (100 nM) (Fig. 1e, green) for 48 hrs. Additionally, AR is expressed in the nucleus of VSMCs, as evidenced by DAPI counterstaining (Fig. 1e, blue, white arrow). Increased expression of AR following treatment with testosterone was also confirmed by western blotting (Fig. 1f,g). RT-PCR analysis revealed aromatase was expressed by mouse ovary and testis, but not in VSMCs (Fig. 1h).

### Androgens induce high P_i_-dependent VSMC calcification

**Figure 2:**
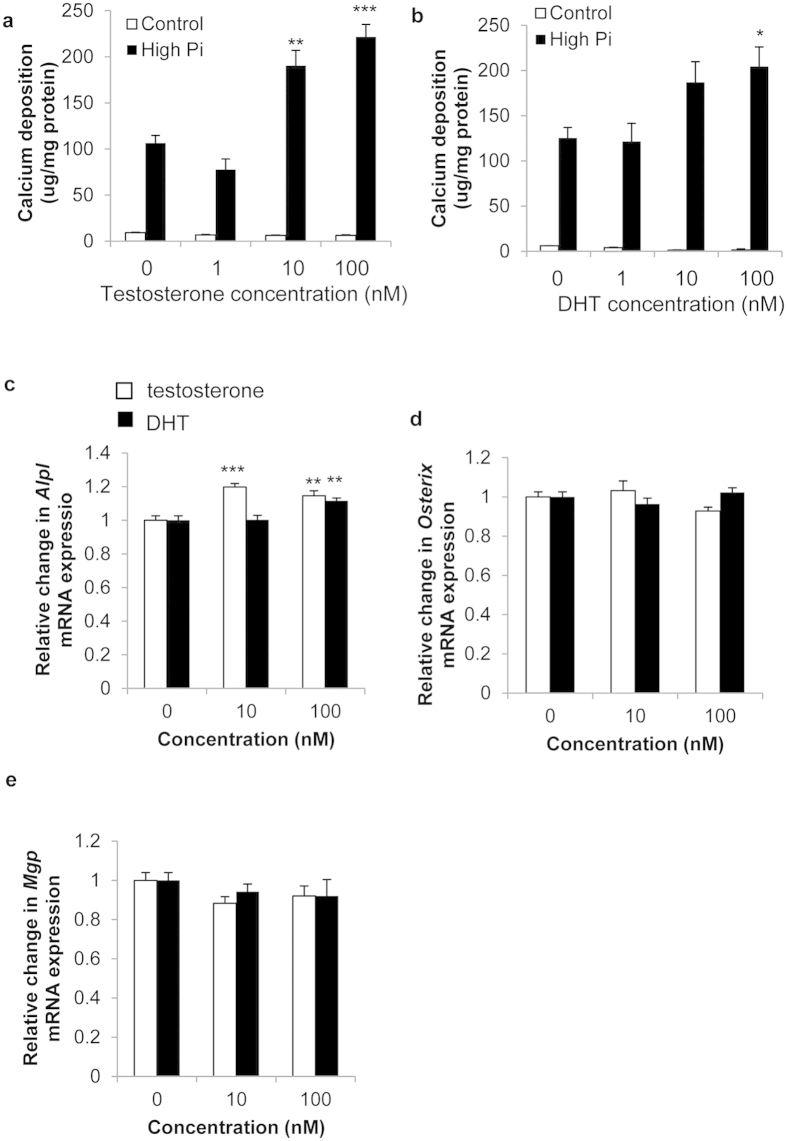
Androgens promote VSMC calcification and regulate osteogenic gene expression. VSMCs were cultured with high phosphate (3 mM P_i_; filled bar) or control (1 mM P_i_; white bar) for up to 9 days. Calcium content (μg/mg protein) of cells treated with (**a**) testosterone (1–100 nM) (n = 4) or (**b**) DHT (1-100 nM) (n = 4). Fold change in the mRNA expression of (**c**) *Alpl* (**d**) *Osterix* and (**e**) *Mgp* following 48 hr treatment with testosterone (white bar) or DHT (filled bar) (n = 6). Results are presented as mean +/− S.E.M *P < 0.05; **P < 0.01; ***P < 0.001 compared with control.

To investigate the role of the AR in VSMCs, we examined the effects of androgens on VSMC calcification. Initial studies examined whether androgens directly regulate the osteogenic differentiation and calcification of VSMCs. Since arterial calcification is highly correlated with elevated serum P_i_ levels, VSMCs were cultured in growth medium containing high P_i_ (3 mM) as previously described^33^,^34^. Cells were treated with 1–100 nM testosterone or DHT in the presence or absence of high P_i_ medium for up to 9 days. A minimum concentration of 10 nM testosterone treatment significantly increased calcium deposition of VSMCs at 9 days, as evaluated by HCl leaching (1.8 fold, P < 0.01, Fig. 2a). A minimum concentration of 100 nM DHT treatment significantly increased calcium deposition in VSMCs at 9 days (1.6 fold, P < 0.05, Fig. 2b). Testosterone and DHT (100 nM) induced a significant increase in the mRNA expression of *Alpl* a key mediator of mineralisation (1.14 fold, P < 0.01) (Fig. 2c). No change in the mRNA expression of the osteogenic marker *Osterix* or the mineralisation inhibitor *Mgp* was observed (Fig. 2d,e).

### Androgens induce calcification through the AR

**Figure 3:**
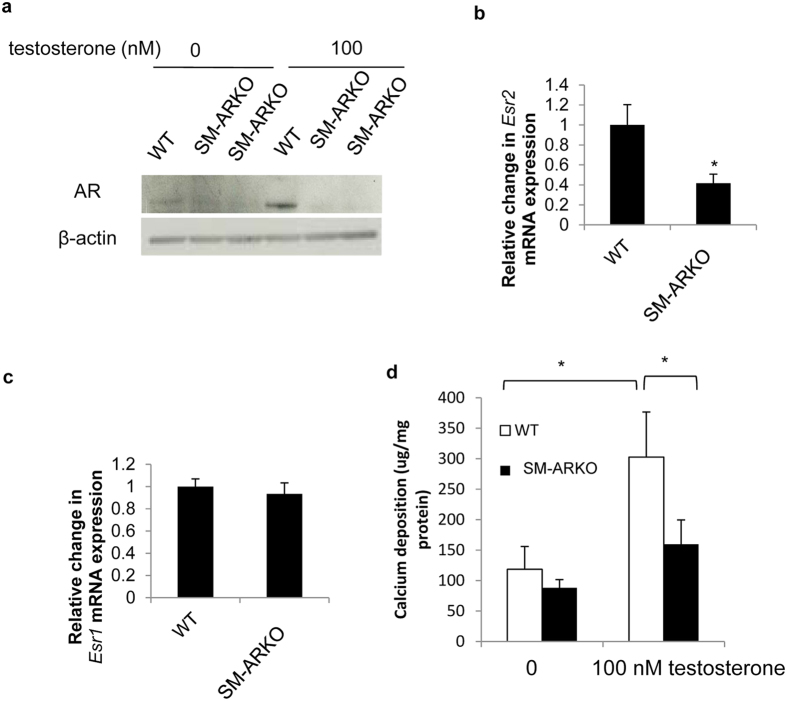
Androgens promote P_i_-induced calcification via the AR. (**a**) AR protein expression in SM-ARKO VSMCs versus WT control cells in the presence or absence of testosterone (100 nM) (Images were cropped from original scans, and gels were performed under the same experimental conditions. Unprocessed original scans are shown in Suppl Fig. 1). Fold change in the mRNA expression of (**b**) *Esr2* and (**c**) *Esr1* in SM-ARKO VSMCs versus WT control cells (n = 5). (**d**) Calcium content (μg/mg protein) of SM-ARKO and WT VSMCs cultured with high phosphate (3 mM P_i_) for 9 days and treated with testosterone (100 nM) (n = 6). Results are presented as mean + /− S.E.M *P < 0.05 compared with control.

**Figure 4:**
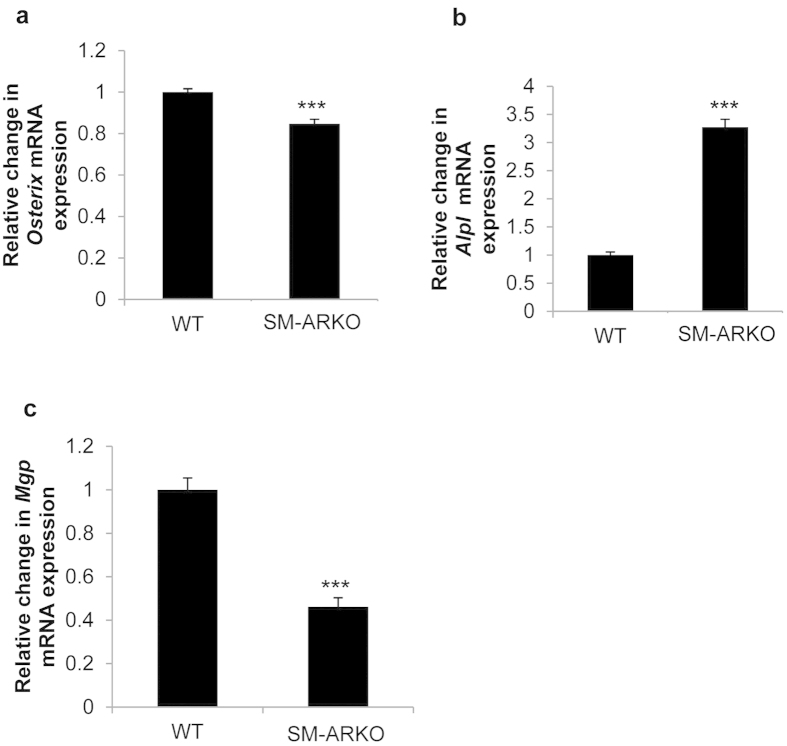
Altered mRNA expression of osteogenic genes in SM-ARKO VSMCs. Fold change in the mRNA expression of (**a**) *Osterix* (**b**) *Alpl* and (**c**) *Mgp* in SM-ARKO VSMCs versus WT control cells (n = 5). Results are presented as mean + /− S.E.M ***P < 0.001 compared with control.

To examine whether testosterone-stimulated VSMC calcification is mediated through the AR, further studies were undertaken using cells derived from VSMC-specific AR-ablated (SM-ARKO) mice. Protein expression studies confirmed AR ablation in the VSMCs (Fig. 3a). Furthermore, testosterone exposure notably up-regulated AR protein expression in WT VSMCs, whereas AR expression remained absent in comparably-treated SM-ARKO cells (Fig. 3a). Additional studies revealed a marked reduction in *Esr2* mRNA expression in SM-ARKO mice versus WT controls (0.4 fold; P < 0.05; Fig. 3b), whereas *Esr1* expression remained unaltered (Fig. 3c). Consistent with our earlier observations (Fig. 2a), 100 nM testosterone significantly increased VSMC calcification in WT cells. However, testosterone-induced calcification was blunted in SM-ARKO cells (0.52 fold; P < 0.05; Fig. 3d). Consistent with these data, SM-ARKO VSMCs showed a significant reduction in *Osterix* mRNA expression (0.85 fold; P < 0.001; Fig. 4a). However, intriguingly, a counter-intuitive increase in *Alpl* was observed (3.3 fold; Fig. 4b, P < 0.001), with a concomitant decrease in the mineralisation inhibitor *Mgp* (0.5 fold; Fig. 4c, P < 0.001).

### Testosterone has no effect on apoptosis of VSMCs

**Figure 5:**
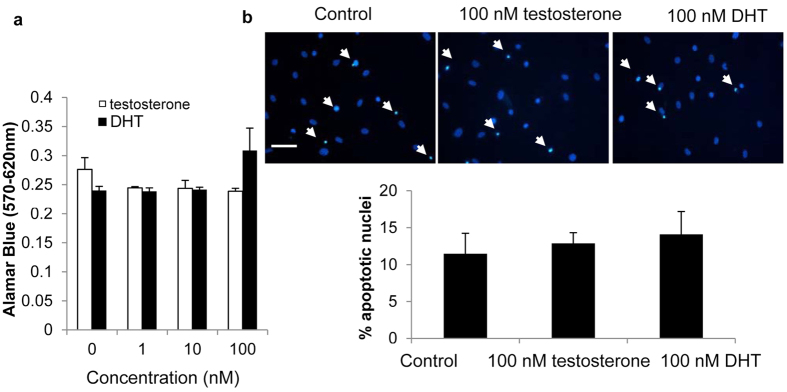
Testosterone has no effect on apoptosis of VSMCs. Testosterone or DHT (100 nM) treatment does not (**a**) reduce VSMC viability (n = 6) or (**b**) induce apoptosis (n = 6).

Apoptosis plays an important role in vascular calcification, we therefore examined the effect of testosterone treatment on VSMC apoptosis. Alamar blue assay showed no effect of either testosterone or DHT on cell viability (Fig. 5a). DAPI staining revealed no effect of either testosterone or DHT on nuclei apoptosis (Fig. 5b).

## Discussion

Whilst testosterone deficiency has been linked with cardiovascular disease in men^1^, the effects of ART on vascular health are currently highly controversial^7^,^8^. Given the increasing pharmacological use of androgens in society, a more complete understanding of the cardiovascular safety of exogenous androgens is essential. This investigation addressed the hypothesis that androgen-induced stimulation of the AR induces arterial calcification. Our work offers new insight into the role of androgens in disease and provides direct evidence to suggest that testosterone contributes to the pathological process of vascular calcification.

Our *in vitro* investigations revealed that exogenous testosterone and DHT treatment both had striking effects on the induction of VSMC calcification. A concomitant increase in expression of *Alpl*, a recognised regulator of osteoblastic differentiation and matrix mineralisation of VSMCs was observed. Indeed, there is a substantial body of evidence linking TNAP elevation with vascular calcification. TNAP upregulation has been observed in calcified coronary atherosclerotic plaques^35^, as well as in medial vascular calcification associated with diabetes^36^, in patients undergoing dialysis^37^,^38^ and has been proposed as a cause of the vessel calcification seen in uremia^39^. Consistent with these findings, an association between long-term anabolic steroid abuse in men and early vascular calcification has been previously reported^40^. Similarly, in women with polycystic ovary syndrome, elevated endogenous androgen levels are associated with increased vascular calcification^41^ and high dose testosterone administration in post-menopausal women has been associated with increased atherosclerosis as measured by detection of calcified deposits^42^. In addition, recent animal studies have employed the Apolipoprotein E mouse model to directly link testosterone and calcification in the vasculature^31^. However, in contrast to these data, a recent *in vitro* study has reported inhibitory effects of androgens on VSMC calcification^32^. This discrepancy may reflect key differences in the species, passage status and culture conditions of the cells investigated.

Androgens act mainly through transcriptional control of target genes mediated by AR^11^. In the present study we confirmed that AR is expressed in VSMCs, and identified for the first time the presence of AR expression in calcified human femoral artery and aortic valve tissue. These data are consistent with previous studies showing expression of AR in smooth muscle cells and endothelial cells in arteries^14^,^43^. Further studies are required to comprehensively establish AR expression levels throughout the different layers of arterial blood vessels. The ability of DHT to replicate the effects of testosterone, together with our data revealing a lack of aromatase expression, suggests that the pro-calcificatory androgenic effects are mediated directly through AR. Consistent with this hypothesis, our *in vitro* studies demonstrated that AR is up-regulated in VSMCs by androgen treatment. This is in agreement with reports of increased local AR expression in the innominate artery in response to testosterone and DHT-induced calcification in the ApoE mouse model^31^. However, in a recent clinical analysis, no association between coronary artery calcification in men and AR expression was observed^13^. Potentially the exclusion of patients with existing coronary artery disease may have resulted in an underestimation of any association between AR expression and calcification in this study.

The utilisation of a novel vascular selective ARKO mouse model was central to this investigation. The employment of a cell-specific mouse line with AR deleted from VSMCs^44^ emphatically highlights, for the first time, a functional role for AR in androgen-induced vascular calcification. Reduced *Osterix* expression was observed in SM-ARKO VSMCs, suggesting androgen receptor may regulate osteogenic differentiation of VSMCs. Unexpectedly, altered expression of the calcification regulator *Mgp* and *Alpl* was also noted in SM-ARKO cells. Further studies are required to investigate their roles in AR-mediated calcification.

This study reveals AR expression in diseased vascular tissue and highlights a potential link between pharmacological androgen use and vascular calcification. We note that this is achieved in a small sample, and these data need to be extended with further samples in future investigations. However, the clear demonstration that androgens directly act through the AR to mediate pro-calcificatory effects in VSMCs may have important health ramifications for patients with elevated AR expression in their vascular tissues and for the use of ART. These data also have strong implications for the role of androgens in aortic valve calcification, which independently predicts mortality in aortic stenosis^45^,^46^.

## Materials and Methods

### Human tissues

Arterial samples were obtained with appropriate ethical approval from a patient undergoing amputation for critical limb ischaemia (Ethics Number: 13/ES/0126). Research ethics committee approval (National Health Service West of Scotland Research Ethics Committee: 12/WS/0227) and the written and informed consent of all participants were obtained. The sample was from an amputated leg from a man who had unsalvagable critical limb ischaemia secondary to *in situ* thrombosis of typical atherosclerotic femoro-popliteal peripheral arterial disease. He was an ex-smoker with no history of any endocrine disorder (including diabetes) and had an entirely normal masculine phenotype. He was on no hormonal treatment but was receiving standard therapy for atherosclerosis, namely aspirin and a HMG-CoA reductase inhibitor (statin). He had normal renal function and so the aetiology of the calcification in his femoral artery was associated with atherosclerosis. Serum testosterone levels were not measured. Human valve tissues were obtained with appropriate ethical approval from patients undergoing valve replacement surgery (Ethics No: 13/ES/0126). The control valve was from a 79 year old woman with aortic incompetence and generalised aortic dilatation with ascending aorta involvement. There was minor to moderate left ventricular impairment. The calcified valve was from a 67 year old woman with severe aortic stenosis detected by echocardiography. The aortic valve was removed at the time of aortic valve replacement operation, and carefully preserved the integrity of the valve architecture. An informed consent was given for the use of the human tissue and this study was performed in conformation with the declaration of Helsinki.

### Materials

The AR antibody (Catalog: SC-816-G) was from Santa Cruz Biotechnology (Paso Robles, US). Testosterone and DHT were obtained from Sigma (Poole, UK). The SMA antibody (Catalog: A2547) was from sigma. DAPI, Alexa Fluor@488 donkey-anti goat antibody (Catalog: A-11055) and Alexa Fluor@594 goat-anti mouse antibody (Catalog: A-11005) were from Invitrogen (Paisley, UK).

### Mice

Mice with selective ablation of AR from VSMCs (SM-ARKO) were generated and genotyped as previously described^14^. All animal experiments were performed under UK Home Office licensed approval in accordance with Directive 2010/63/EU of the European Parliament and were maintained in accordance with Home Office guidelines for the care and use of laboratory animals. C57BL/6 mice were supplied by Charles River Laboratories (Harlow, Essex, UK).

### Isolation of primary murine VSMCs

Mice were euthanized by cervical dislocation. Primary murine VSMCs were isolated as previously described^25^,^33^,^34^. After removal of adventitia, the aorta was cut open to expose the endothelial layer under a dissection microscope. Tissues from eight animals were pooled and incubated with 1 mg ml^−1^ trypsin (Invitrogen) for 10 min in order to remove any remaining adventitia and endothelium. After a further overnight incubation at 37 °C in a humidified atmosphere of 95% air/5% CO_2_ in α-MEM medium (Invitrogen) supplemented with 10% Fetal Bovine Serum (Invitrogen) and 1% gentamicin (Invitrogen), tissues were digested with 425 U/ml collagenase type II (Worthington Biochemical Corporation, Lakewood, USA) for 5 hrs. Medial cells were released and cell suspensions were centrifuged at 2000 g for 5 min. The cell pellet was washed and resuspended in the above mentioned culture medium. Isolated VSMCs were cultured with growth medium for two passages in T25 tissue culture flasks (Greiner Bio-one, GmbH, Frickenhausen, Baden-Wurttemberg, Germany) coated with 0.25 μg/cm^2^ laminin (Sigma) to promote maintenance of the contractile differentiation state^47^.

### Induction of calcification

Primary VSMCs were seeded in growth medium at a density of 1.5 × 10^4^/cm^2^ in multi-well plates. Calcification was induced as previously described^25^,^33^,^34^. In brief, cells were grown to confluence (day 0) and switched to calcification medium, which was prepared by adding 1 M inorganic phosphate (P_i_) (a mixture of NaH_2_PO_4_ and Na_2_HPO_4_, pH 7.4) (Sigma), to reach a final concentration of 3 mM P_i_. VSMCs were incubated for up to 9 days in 95% air/ 5% CO_2_ and the medium was changed every third/fourth day. Testosterone (1–100 nM) or DHT (1–100 nM) were added at day 0, according to previous publications^14^,^32^.

### Determination of calcification

Calcium deposition was quantified as previously described^34^,^48^,^49^. Briefly, cells were rinsed twice with phosphate buffered saline (PBS) and decalcified with 0.6 N HCl at room temperature for 24 hrs. Free calcium was determined colorimetrically by a stable interaction with phenolsulphonethalein using a commercially available kit (Randox Laboratories Ltd., County Antrim, UK) and corrected for total protein concentration (Bio-Rad Laboratories Ltd, Hemel Hempstead, UK).

### Analysis of gene expression

RNA was extracted using RNeasy total RNA (Qiagen, West Sussex, UK), according to the manufacturer’s instructions. RNA was reverse transcribed and specific cDNAs were semi-quantified by end point PCR or quantified by real-time PCR using the SYBR green detection method as previously reported^33^,^34^,^48^ Primers were obtained from Eurofins MWG Biotech (Ebersberg, Germany), Qiagen, Ambion (Cambridge UK) and Primer Design (Southampton, UK), with available sequences provided in the online Data Supplement (Suppl. Table S1).

### Alamar blue staining

VSMCs were grown on 96-well plates at a density of 5,000 cells/ well. Alamar blue assay was performed as previously described^49^. Alamar blue reagent (Invitrogen) (10 μl/100 μl medium) was added to culture medium for the final 4 hrs of culture at 37 °C, 5% CO_2_. Optical density absorbance was analysed at wavelengths of 570 nm and 620 nm using a spectrophotometer (Multiskan Ascent; Thermo Electron Corporation,Vantaa, Finland).

### Apoptosis

Apoptotic VSMCs were determined by manually counting pyknotic nuclei after staining with DAPI (Invitrogen) as previously described^50^.

### Fluorescent immunocytochemical staining

VSMCs were seeded on glass coverslips in 12-well plates at a density of 50, 000 cells/well. Following confluence, VSMCs were serum-restricted for 24 hrs and treated with contro or 100 nM testosterone for 48 hrs. Cells were fixed with 4% paraformaldehyde and washed with PBS. The fixed cells were permeabilised with 0.3% Triton-X 100 (Sigma) and incubated with anti-AR antibody (Santa Cruz Biotechnology) and anti-SMA (Sigma) overnight at 4^o^ C. After washing, cells were incubated with Alexa Fluor@488 donkey-anti goat antibody and Alexa Fluor@594 goat-anti mouse antibody (Invitrogen) for 1 hr in the dark. Glass coverslips were mounted onto slides with Prolong®Gold Anti-Fade Reagent contained DAPI (Invitrogen). Fluorescence signal was detected under a Leica fluorescence microscope (Milton Keynes, UK).

### Western analysis

VSMCs were treated with 100 nM testosterone for 48 hrs. Cells were lysed in PhosphoSafe extraction buffer (Merck Biosciences Ltd, Nottingham, UK) containing “Complete” protease inhibitor cocktail (Roche, East Sussex, UK) according to manufacturer’s instructions. Western blotting was performed as previously described^23^,^32^,^33^. Nitrocellulose membranes were probed overnight at 4 °C with anti-AR antibody (Santa Cruz Biotechnology), washed in TBST and incubated with anti-goat IgG-peroxidase respectively (DAKO, Glostrup, Denmark) for 1 hr (1:1000 dilution in 5% milk). The immune complexes were visualised using enhanced chemiluminescence (ECL) (GE Healthcare, Buckinghamshire, UK). Membranes were then washed in ‘stripping buffer’ (Pierce, Rockford, Il, USA) and re-probed for 1 hr for β-actin expression (1:5000 dilution in 5% milk; anti β-actin clone AC15; Sigma-Aldrich).

### Histology and immunohistochemistry

Tissues were fixed in 10% neutral buffered formalin (NBF) for 24 hrs before being dehydrated and embedded in paraffin wax and sectioning at 4 μm using standard procedures. Sections were de-waxed in xylene and stained with Von Kossa and alizarin red (Sigma) to visualise phosphate and calcium deposition, respectively. Immunohistochemistry was performed using the VECTASTAIN ABC Kit (Goat IgG) (Vector Labs, Peterborough, UK) according to manufacturer’s instructions. Sections were de-waxed in xylene and de-masked with citric acid based antigen unmasking solution (Vector Labs). Endogenous peroxidase and non-specific antibody binding were blocked before overnight incubation at 4 ^o^C with 2 μg IgG/ml anti-AR antibody (Santa Cruz Biotechnology). The sections were then washed in PBS, incubated with diluted biotinylated secondary antibody (1:200 dilution) for 30 min. After washing in PBS for 5 min, the sections were incubated for 30 min with VECTASTAIN® ABC Reagent (Vector Labs). The sections were then incubated with DAB substrate reagent (0.06% DAB, 0.1% H_2_O_2_ in PBS) until the desired stain intensity developed. The sections were finally dehydrated, counterstained with haematoxylin and eosin and mounted in DePeX. Control sections were incubated with non-immune goat IgG (2 μg IgG/ml) in place of the primary AR antibody.

### Statistical analysis

General Linear Model analysis and the Students t-test were used to assess the data. All data are expressed as the mean + /− S.E.M. Statistical analysis was performed using Minitab 16. P < 0.05 was considered to be significant.

## Additional Information

**How to cite this article**: Zhu, D. *et al.* Ablation of the androgen receptor from vascular smooth muscle cells demonstrates a role for testosterone in vascular calcification. *Sci. Rep.* **6**, 24807; doi: 10.1038/srep24807 (2016).

## Supplementary Material

Supplementary Information
