# Supplementary material for: Ablation of the androgen receptor from vascular smooth muscle cells demonstrates a role for testosterone in vascular calcification
Source: Sci Rep. 2016 Apr 20;6:24807. doi: 10.1038/srep24807 (PMC4837411; doi:10.1038/srep24807)

Ablation of the androgen receptor from vascular smooth muscle cells demonstrates a role for testosterone in vascular calcification

Dongxing Zhu[1*], Patrick W. F. Hadoke[2], Junxi Wu[2,4], Alex T. Vesey[2], Daniel Lerman[3], Marc R. Dweck[2], David E. Newby[2], Lee B. Smith[4], Vicky E. MacRae[1]

Suppl. Table S1

Gene	Primer sequence
Alpl	Forward : 5' GGG ACG AAT CTC AGG GTA CA 3'
	Reverse :5' AGT AAC TGG GGT CTC TCT CTT T 3'
Mgp	Forward : 5' GTG GCAACC CTG TGC TAC 3'
	Reverse :5'CAG GCT TGT TGC GTT CC 3'
18S	**Ambion, Huntingdon, Cambs, UK, sequence not disclosed***
Gapdh	**Unknown, purchased commercially from Primer Design.***
Osterix	**Unknown, purchased commercially from Qiagen.***
Esr1	
Esr2	
Aromatase	

Supplementary Table 1: Primer pairs used for PCR analysis.

Suppli. Fig 1

Fig.1f

AR

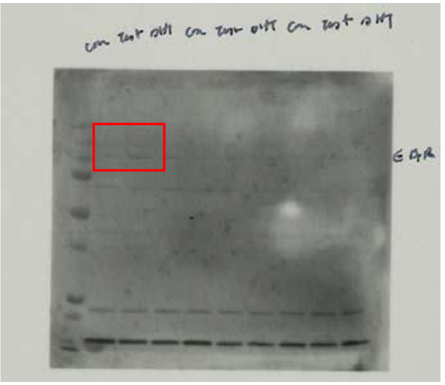

β-actin

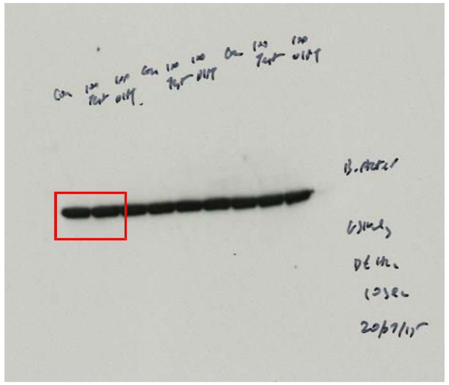

Fig.1h

Aromatase

18s

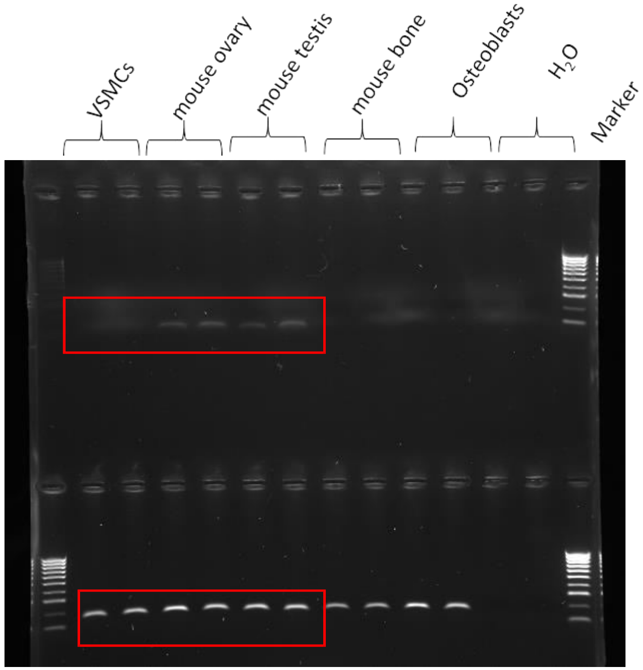

Fig.3a

AR

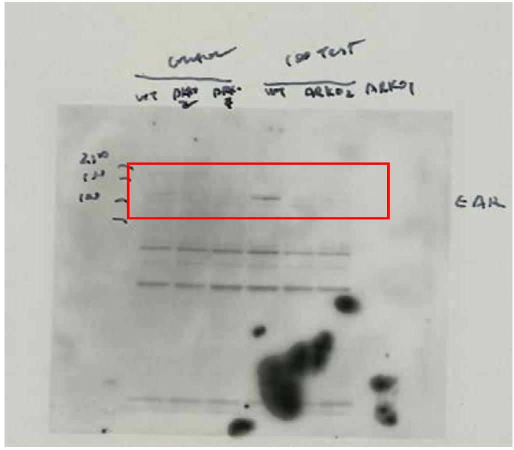

β-actin

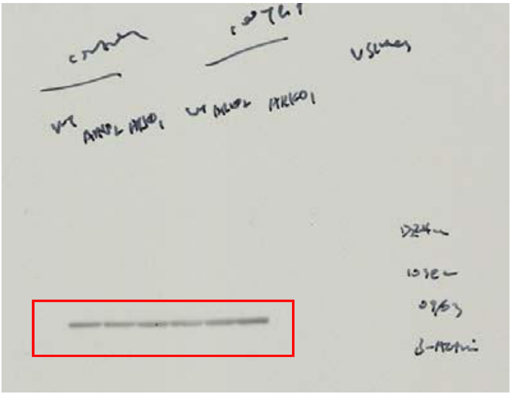

Supplement: Supplementary Information [file srep24807-s1.pdf]
